# Relationship Between Sunlight Exposure, Erythemal Dose, and Vitamin D Concentrations in Adolescents: A Cross-Sectional, Multi-center Study

**DOI:** 10.7759/cureus.96822

**Published:** 2025-11-14

**Authors:** Chidvilas More, Neha Kajale, Vivek Patwardhan, Ketan Gondhalekar, Sharvani Patil, Aboli Bhalerao, Karishma Bhade, Niharika Shrivastava, Anuradha Khadilkar

**Affiliations:** 1 Department of Growth and Pediatric Endocrinology, Hirabai Cowasji Jehangir Medical Research Institute, Pune, IND; 2 School of Health Sciences, Savitribai Phule Pune University, Pune, IND

**Keywords:** adolescents, deficiency, ed, erythemal dose, india, poly sulphone badges, psu, sunlight exposure, vitamin d, vit. d

## Abstract

Background

Vitamin D (Vit. D) plays a vital role in bone health and calcium-phosphorus homeostasis. Its deficiency is a major global health concern, affecting populations even in sun-rich countries like India. Indian adolescents, predominantly urban dwellers and girls, show a higher deficiency of Vit. D. Objective assessment of sunlight exposure in the Indian pediatric population in relation to Vit. D status is limited.

Objectives

This study examined the association between Vit. D concentrations, sunlight exposure (as measured using Polysulphone (PSU) dosimeter badges), and anthropometric parameters among urban and rural Indian adolescents across six states. The study investigated the differences in Vit. D status with changes in seasons, sex, and geographical regions.

Methods

A large-scale, multi-center research project that included 2500 children and their reported Vit. D prevalence was conducted from July 2016 to October 2017. This cross-sectional study was conducted in six Indian states across urban and rural settings. The study took place during the winter and rainy seasons. Vit. D status was determined using a previously standardized liquid chromatography-mass spectrometry (LC-MS)/MS-based method to measure 25-hydroxy-vitamin D3 (25(OH)D3) from dried blood spots, as 25(OH)D accounts for over 90% of the total 25(OH)D3 concentration in Indians. In the current study, we report data on a subset of 545 children who wore PSU badges on their wrists. Anthropometric data - height, weight, and BMI - were collected following standard protocols. Vit. D concentrations were measured from dried blood spots. Sunlight exposure was quantified as standard erythemal dose (ED) using PSU badges. Statistical analyses of the data included correlation, Mann-Whitney U tests, and multiple regression.

Results

Urban children were taller, heavier, and had lower Vit. D concentrations, sunlight exposure, and ED than rural children (p < 0.05). Rural boys had the highest Vit. D concentrations (24.6 ng/ml). Vit. D had a significant negative correlation with weight in urban girls (p < 0.05). Significant negative correlation (p < 0.05) was found between BMI, waist circumference, and Vit. D concentrations, and a positive association was found between ED and Vit. D concentrations in urban areas. There was a strong positive correlation between ED, sunlight exposure, and Vit. D concentrations (p>0.05). The winter season and rural settings saw notably higher ED as compared to the rainy season and the urban setting.

Conclusion

Sunlight exposure was a significant predictor of Vit. D status in Indian adolescents. Rural residence and winter season were associated with higher sunlight exposure and better Vit. D status. Girls were particularly vulnerable to Vit. D deficiency. These findings highlight the need for targeted interventions, especially in urban areas and among girls, to improve sunlight exposure and Vit. D status. Policy efforts should consider promotion of outdoor activity, with particular emphasis on improving Vit. D status during the monsoon season.

## Introduction

Vitamin (Vit.) D has a crucial role in maintaining calcium and phosphorus homeostasis and has effects on bone, bone cells, and growth plates [1]. It increases the efficiency of the intestinal absorption of dietary calcium, reduces calcium losses in urine, and mobilizes calcium stored in the skeleton. Human skin synthesizes Vit. D when exposed to ultraviolet B (UVB) radiation. Factors like clothing, sunscreen use, intentional avoidance of sunlight, and residence at high latitudes can significantly reduce its production. Globally, more than one billion people suffer from Vit. D deficiency with an undiagnosed condition [2]. Vit. D is an essential micronutrient for multiple physiological processes, and its deficiency, along with insufficiency, affects individuals of all ages, even in countries with abundant sunlight or where fortification efforts have been in place for years, and remains a worldwide public health problem that cannot be ignored [3]. The primary cause of this is a lack of awareness that moderate sun exposure is the main source of Vit. D for most people [4]. In fact, less than 50% of the global population maintains adequate Vit. D levels, particularly during the winter months, making Vit. D deficiency a global environmental health problem [5].

In India, approximately 490 million individuals are deficient in Vit. D, out of which 31% are children and adolescents [6-8]. Clothing type, use of sunscreen, sex, residence, and season also affect the status of Vit. D concentrations. Vit. D deficiency is common in rural Indians despite adequate sunlight exposure [9]. However, there is limited research on objective measures of sunlight exposure and their impact on Vit. D levels, especially in the Indian pediatric population. Studies using self-reported data, UVB levels, and time spent outdoors often face a reporting bias [10,11]. To address this gap, polysulphone (PSU) dosimeter badges are used, as they are sensitive to UVB radiation, which is responsible for Vit. D production in the skin [8]. Significant correlations between measures of UVB exposure by dosimeters and self-reported sunlight exposure have been reported by many studies [12-14]. However, very few studies in India have objectively studied sunlight exposure and the relationship between Vit. D concentrations and sunlight exposure.

Objectives

This study aimed to objectively measure sunlight exposure as erythemal dose (ED) and to assess the relationship between Vit. D concentrations and sunlight exposure (measured using PSU badges). Specific objectives were: 1) To assess the relationship of Vit. D concentrations with sunlight exposure (as ED assessed by PSU badges) and anthropometric parameters in urban and rural adolescents; 2) To assess the ED received as per place of residence, sex, and season.

## Materials and methods

We conducted a multicentric, cross-sectional, observational school-based study in six states of India, viz. Maharashtra, Gujarat, Chhattisgarh, Assam, Tamil Nadu, and Punjab. The study took place during the winter and rainy seasons (from July to February), as the summer months coincide with school holidays [10]. Vit. D status was determined using a previously standardized liquid chromatography-mass spectrometry (LC-MS)/MS-based method to measure 25-hydroxy-vitamin D3 (25(OH)D3) from dried blood spots, as 25(OH)D3 accounts for over 90% of the total 25(OH)D concentration in Indians [13,15,16].

The current study built upon a larger research project conducted from July 2016 to October 2017, which included 2500 children and reported on Vit. D prevalence. Multi-stage stratified random sampling was performed. Six Indian states were randomly selected, covering the wide geographical areas of the country. One city and a nearby village were randomly selected from each of these states. The selected cities and villages were Pune-Ranjangaon (18.5°N), Bilimora-Gandevi (20.7°N), Raipur-Kurud (21.2°N), Diphu-Manja (25.8°N), Chennai-Urapakaam (13.0°N), and Mohal-Lalru (30.7°N). A list of schools from the selected centers was made, and out of the 100 schools approached, the 40 schools that gave permission were included in the study after obtaining necessary permissions from the institutions, health authorities, and parents (schools mainly disagreed because they could not invest the time required for the study). Written consent and assent were obtained from participants older than 7 years, with ethics committee approval granted on June 21, 2016 [10].

In the current study, we report data on a subset of 545 children who were provided with PSU badges and whose data were retrieved. All children were examined by pediatricians, and their medical records were reviewed. Children suffering from chronic disorders or disorders likely to affect calcium and vitamin D metabolism, and those receiving calcium or vitamin D supplements, were excluded, and others were included. Height, weight, and BMI were collected following standard protocols. The formula for calculating BMI was: weight (kg) / height (m²), and anthropometric measurements were converted into Z-scores using Indian reference data [14]. Bioelectrical impedance analysis (BIA) was used to measure body composition, with fat percentage and lean mass Z-scores calculated using reference data [16]. Sunlight exposure was measured using PSU dosimeter badges, which participants wore on a leather bracelet from sunrise to sunset on a typical weekday. The badges were returned after one week for analysis at the University of Manchester. The resulting exposure data was reported in standard erythema doses (SED), where 1 SED = 100 J/m². Dried blood spots were collected before the use of PSU badges using sterile safety lancets and Whatman filter paper no. 903, with samples stored at -80 °C for further analysis [10].

PSU data collection

Within one week of enrolment and collection of the blood samples, individuals were asked to wear the bracelets with PSU dosimeters badges with dorsally facing PSU film, on a typical school day from sunrise to sunset. Instructions were given to them to store the dosimeter in the supplied envelope (prepared from thick and dark material to prevent further UVB exposure during storage) and return it within a week. When all exposed badges were returned by participants, they were sent to the laboratory at the University of Manchester for analysis, and the resulting individual badge doses were reported in SED units. We could retrieve the PSU data of 545 children only.

Data entry and statistical analysis

It involved entering all data twice into MySQL (https://www.mysql.com/) and trapping all the errors using range checks. Measures of central tendency, ranges, and distributions of continuous variables were used to examine the consistency of the dataset. To identify outliers and data inconsistencies for categorical variables, frequency tables were created. SPSS software (version 26.0; IBM Corp., Armonk, NY, US) was used for data analysis. The Mann-Whitney U test and correlational analysis were performed; further, multiple regression was performed to define predictors of Vit. D concentrations. In the regression analysis, Vit. D concentration was the dependent variable, while ED, residence, sex, season, age, BMI, body fat, height, and weight were the independent variables. Age, sex, and residence were the controlled variables. Statistical significance was interpreted using the p-value. If the p-value is less than or equal to 0.05 (p 0.05), the result is considered statistically significant, i.e., it is unlikely to have occurred by random chance. No multiple comparisons were performed.

## Results

The mean age of urban children was 12.9 years, and that of rural children was 13.5 years. Urban children were taller, heavier, and had lower Vit. D concentrations, sunlight exposure, and ED than rural children (p < 0.05). Rural boys had the highest lean body mass Z-score (1.16) and the highest Vit. D concentrations (24.6 ng/ml) (Table 1).

**Table 1:** Descriptive statistics of the study participants Values are in median (lower quartile and upper quartile): a: significant difference between urban boys and rural boys by the Mann-Whitney test (p<0.05) b: significant difference between urban girls and rural girls by the Mann-Whitney test (p<0.05) c: significant difference between urban boys and urban girls by the Mann-Whitney test (p<0.05) d: significant difference between rural boys and rural girls by the Mann-Whitney test (p<0.05)

Variables	Urban		Rural	
	Boys (n=178)	Girls (n=145)	Boys (n=97)	Girls (n=125)
Height (cm) ^a, b^	154.1 (144.1, 163.4)	153.0 (147.0, 157.0)	150.1 (139.7, 157.1)	149.2 (144.9, 153.5)
Height Z-Score ^a, b, c, d^	0.1 ( -0.6, 0.6)	0.3 (-0.4, 0.7)	-0.7 ( -1.5, -0.1)	-0.5 (-1.2, 0.2)
BMI (kg/m²) ^a, d^	17.5 (15.7, 20.3)	17.1 (15.5, 19.2)	16.1 (14.7, 17.6)	17.5 (15.8, 19.2)
BMI Z score ^a, c, d^	-0.3 ( -0.8, 0.4)	-0.5 (-1.1, 0.2)	-0.8 ( -1.2, -0.3)	-0.5 (-1.0, 0.0)
Lean body mass Z score^ a,b,d^	0.4 (-0.5, 1.0)	0.2 ( -0.4, 0.7)	1.2 (0.6, 1.5)	0.5 (-0.1, 1.1)
Fat Z score^ a, b, d^	-0.4 (-1.0, 0.5)	-0.2 (-0.7, 0.4)	-1.1 (-1.6, -0.5)	-0.4 (-1.0, 0.1)
Vit. D (ng/ml)^ a, b, c^	18.7 (13.9, 25.8)	17.2 (11.9, 22.2)	24.6 (17.6, 32.4)	22.3 (16.5, 27.8)
Vit. D group ^a, b, c^				
Deficient	29.0 (16.3%)	37.0 (25.5%)	8.0 (8.2%)	10.0 (8.0%)
Insufficient	65.0 (36.5%)	57.0 (39.3%)	24.0 (24.7%)	46.0 (36.8%)
Sufficient	84.0 (47.2%)	51.0 (35.2%)	65.0 (67.0%)	69.0 (55.2%)
Erythemal dose^ a, b, c^	0.8 (0.4, 1.1)	0.5 (0.3, 0.8)	0.8 (0.5, 1.2)	0.7 (0.5, 1.1)

**Figure 1:**
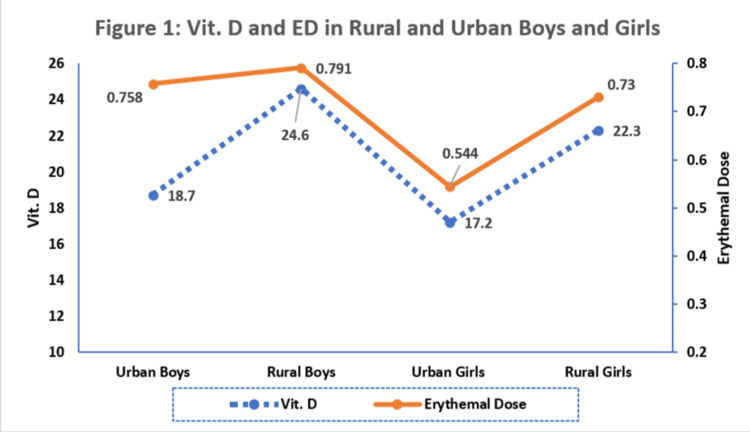
Vit. D and erythemal dose (ED) in rural and urban boys and girls

Urban children had lower Vit. D concentrations and ED than rural children. Girls had a higher prevalence of Vit. D deficiency, with lower ED and Vit. D concentrations compared to boys (Figure 1).

**Figure 2:**
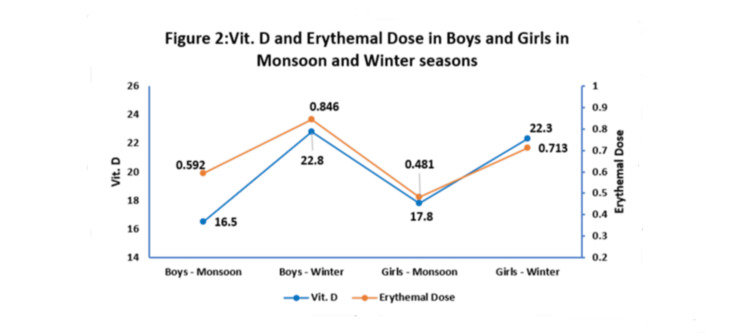
Vit. D and erythemal dose in boys and girls in the monsoon and winter seasons

A prominently high ED was recorded among boys during the winter season. Vit. D prevalence was higher during winter and lower during the monsoon season (Figure 2).

There was a strong positive correlation between ED, sunlight exposure, and Vit. D concentrations (p < 0.05). Weak and mostly statistically insignificant correlations were found between Vit. D levels and anthropometric measurements for urban and rural children. Borderline significant correlation was seen in urban girls and BMI (Table 2).

**Table 2:** Vitamin D correlation matrix *p value < 0.05 is considered significant. Vitamin D has a significant negative relation with weight in urban girls (p < 0.05).

Anthropometric Measurements	Vitamin D (ng/ml)							
	Urban				Rural			
	Boys (n=178)		Girls (n=145)		Boys (n=97)		Girls (n=125)	
									Correlation Coefficient	P Value	Correlation Coefficient	P Value	Correlation Coefficient	P Value	Correlation Coefficient	P Value
	Weight Z score	0.032	0.669	-0.203	0.014*	-0.099	0.333	0.046									0.607
									Height Z score	-0.074	0.327	-0.088	0.294	-0.143	0.163	0.083		0.36
BMI Z score	0.094	0.212	-0.161	0.053	-0.004	0.972	0.035	0.702										

Urban and rural girls showed lower Vit. D concentrations (as measured by ED) than boys (p = 0.127 in urban and 0.499 in rural), but the difference did not reach statistical significance (Table 3). Significant negative association was found between BMI, waist circumference, and Vit. D concentrations, and a positive association was found between ED and Vit. D concentrations in urban areas (p < 0.05) while no such relationship was seen in rural children (Table 3). Girls appeared to have lower Vit. D concentrations than boys, but the difference did not reach statistical significance (p > 0.05). The winter season and rural settings saw notably higher ED (median = 0.89, p = 0.020) as compared to the rainy season and urban setting (median = 0.53, p = 0.011). The negative and low R2 values imply the lack of significant association found between BMI, waist circumference, and Vit. D concentrations as well as between ED and Vit. D concentrations in rural children. Std.ß is the effect size. The 95% confidence intervals suggest that in urban groups, factors like BMI, waist circumference, and erythemal dose have statistically reliable effects on Vit. D status. In contrast, for rural participants, the wide confidence intervals indicate no strong or consistent relationship (Table 3).

**Table 3:** Multiple regression analysis for predictors of Vitamin D concentrations ED: erythemal dose; SED: standard erythema dose *p value < 0.05 is considered significant

Residence	Urban			Rural			Urban			Rural		
Variable	Std.ß	95% CI	P – Value	Std.ß	95% CI	P – Value	Std.ß	95% CI	P – Value	Std.ß	95% CI	P – Value
Age	0.047	(-0.368, 0.743)	0.506	-0.013	(-0.657, 0.577)	0.898	0.172	(0.02, 1.348)	0.044*	0.028	(-0.757, 0.929)	0.84
BMI	-0.226	(-1.337, 0.136)	0.016*	-0.041	(-1.083, 0.778)	0.747	-0.126	(-1.569, 0.748)	0.486	-0.117	(-1.9, 1.023)	0.554
Waist circumference	0.33	(0.574, 2.263)	0.001*	-0.011	(-1.228, 1.135)	0.938	---		---	---		---
Fat Z scores	---			---			0.115	(-0.118, 0.252)	0.476	0.087	(-0.147, 0.25)	0.609
ED (SED)	0.142	(0.017, 0.19)	0.019*	-0.152	(-0.205, 0.007)	0.068	0.133	(0.009, 0.186)	0.031*	-0.149	(-0.206, 0.011)	0.079
Sex (Female)	-0.108	(-0.248, 0.013)	0.077	-0.05	(-0.216, 0.122)	0.581	-0.102	(-0.255, 0.032)	0.127	-0.06	(-0.223, 0.109)	0.499
Adjusted R²	6.40%			-0.60%			2.90%			-0.80%		

## Discussion

This study assessed the relationship between Vit. D concentrations and sunlight exposure in urban and rural children from various regions of India. Urban children were taller, heavier, and had lower Vit. D concentrations, sunlight exposure, and ED than rural ones. Girls had a higher prevalence of Vit. D deficiency, with lower ED and Vit. D concentrations compared to boys. Winters and rural settings saw notably higher ED. The study was conducted only in the schools that gave permission. The study was to collect the Vit. D levels as recorded by PSU badges and as measured by dried blood samples. The study was not conducted in the summer season, as schools have holidays on those days, which makes it extremely difficult to keep track of them. As the Vit. D levels are measured by PSU badges, the typical sun avoidance behavior was not seen in the participants. Data on dietary differences were not collected.

Vit. D deficiency is a global phenomenon. Despite the high ambient UV radiation, approximately one quarter of the population of Australia is estimated to be Vit. D deficient [17]. Prevalence of deficiency has been reported to be <20% in Northern Europe, 30-60% in Western, Southern, and Eastern Europe, and up to 80% in Middle Eastern countries [18,19]. Important determinants reported are skin type, sex, clothing, nutrition, food fortification, supplement use, BMI, sun exposure, use of sunscreen, physical activity, socio-economic status, and degree of urbanization [20]. It was believed previously that people in South Asian countries had sufficient Vit. D concentrations due to enough sunlight, but insufficient Vit. D levels have been reported in these countries, too. Throughout the world, people mostly avoid sunshine for various reasons, resulting in a high prevalence of Vit. D deficiency, even in sunny climates [21]. About 85% Indian population suffers from various degrees of Vit. D deficiency. Vit. D levels in South Indians are relatively higher than those in North Indians due to equatorial proximity [22].

We found in the current study that the ED was higher in rural as compared to urban adolescents. This increased sunlight exposure might be a contributing factor to the higher Vit. D concentrations in rural children. Urban boys and girls had significantly higher Vit. D deficiency than their rural counterparts. The higher ED correlates with higher Vit. D concentrations, suggesting that more sun exposure leads to better Vit. D status. In line with our results, in Bangladesh, 32% of the rural population and 70.6% of the urban population showed Vit. D deficiency indicating higher Vit. D concentrations in rural participants compared to the urban ones. Significant differences in sun exposure, types of clothing, and levels of air pollution of urban and rural settings are responsible for this difference [23]. A study from Central Africa also reports higher levels of Vit. D in rural populations than in urban [24].

We also found that ED and BMI emerged as significant predictors of Vit. D concentrations in the urban group. Rural boys had the highest lean body mass and Vit. D concentrations (mean: 24.6 ng/ml), suggesting a positive relationship between Vit. D concentrations and lean body mass. Urban girls had the lowest lean body mass and Vit. D concentrations, suggesting a potential negative relationship between Vit. D deficiency and lean muscle mass. An interesting contrast was seen between Vit. D concentrations and BMI across urban and rural groups; higher Vit. D concentrations may contribute to healthier body composition, as seen in rural boys with lower BMI. In southern China, girls, adolescents, and urban residents, along with the semi-arid and sub-humid population, showed higher Vit. D deficiency [25]. In urban Beijing, higher to severe prevalence of Vit. D deficiency was found in females than in males [26].

In the current study, it was seen that ED and Vit. D were significantly higher in winter as opposed to the rainy season due to increased availability of sunlight in winter. In Yunan province of China, lower Vit. D levels were observed in spring and winter than in summer and autumn [27]. In Central China, children aged 13-18 years had the highest Vit. D deficiency rate (64.1%) [28]. Globally, too, Vit. D deficiency is more in winter. In Western Siberia, low values of Vit. D were noted in the winter and spring [29]. In North India, the level of Vit. D deficiency in the rainy season was significantly higher as compared to winters and summers [30].

The strengths of our study are that we collected data in adolescents from six states of India and all assessments, including Vit. D assessments, were performed by the same study team. Sunlight exposure using objective methods like the PSU badges has been performed in very few studies. However, although the study collected extensive data, data collection could not be conducted in the summer season due to prolonged school holidays. Also, since this was a multicentric school-based study, Vit. D was assessed using dried blood spots, and other blood parameters could not be assessed. Further, due to the coronavirus epidemic, Vit. D estimations were delayed, resulting in a delay in reporting these results.

## Conclusions

To conclude, ED and BMI had a significant impact on Vit. D concentrations in adolescents, particularly in urban areas. Girls across most regions were found to have a higher prevalence of Vit. D deficiency compared to boys. There was a natural limitation on the sunlight exposure during the monsoon season. Reduced sunlight exposure (with low ED) was a significant factor contributing to higher rates of Vit. D deficiency. The winter season and rural settings saw notably higher ED. Thus, season and geographical residence played an important role in sun exposure and Vit. D concentrations. Thus, increasing sunlight exposure, particularly during the monsoon season, in urban areas and among girls is critical for addressing Vit. D deficiency. Children, including both sexes, should be encouraged to play in sunlight for an ample amount of time during summer holidays. Schools should make sure that all students play in direct sunlight during break time. The period of break time can be increased if necessary. Adolescents, especially urban girls, should be made aware about the fact that sunlight exposure is important for Vit. D levels, it is perfectly all right to get tanned due to sunlight, and it’s important not to avoid sunlight. Awareness about the consumption of milk and milk products, as well as fish and egg yolk, has to be raised. The novelty of PSU dosimeter badges lies in their use of PSU polymer film as a stable, passive UV/radiation-sensitive detector, which combines simplicity, durability, low cost, and ease of optical readout, making them highly practical for personal dosimetry in workplaces, research, and environmental monitoring.
